# Concomitant *EML4-ALK* rearrangement and *EGFR* mutation in non-small cell lung cancer patients: a literature review of 100 cases

**DOI:** 10.18632/oncotarget.17431

**Published:** 2017-04-26

**Authors:** Giuseppe Lo Russo, Martina Imbimbo, Giulia Corrao, Claudia Proto, Diego Signorelli, Milena Vitali, Monica Ganzinelli, Laura Botta, Nicoletta Zilembo, Filippo de Braud, Marina Chiara Garassino

**Affiliations:** ^1^ Department of Medical Oncology, Fondazione IRCCS Istituto Nazionale dei Tumori, Milano, Italy; ^2^ Department of Preventive and Predictive Medicine, Evaluative Epidemiology Unit, Fondazione IRCCS Istituto Nazionale Tumori, Milano, Italy

**Keywords:** EML4-ALK rearrangements, EGFR mutations, non-small cell lung cancer, concomitant, coexistent

## Abstract

The discovery of *EGFR* mutations and *EML4-ALK* gene rearrangements has radically changed the therapeutic *scenario* for patients with advanced non-small cell lung cancer. *ALK* and *EGFR* tyrosine-kinase inhibitors showed better activity and efficacy than standard chemotherapy in the first and second line treatment settings, leading to a clear advantage in overall survival of advanced non-small cell lung cancer patients harboring these genetic alterations.

Historically the coexistence of *EGFR* mutations and *EML4-ALK* rearrangements in the same tumor has been described as virtually impossible. Nevertheless many recent observations seem to show that it is not true in all cases.

In this review we will discuss the available literature data regarding this rare group of patients in order to give some suggestions useful for their clinical management. Furthermore we report here two cases of concomitant presence of both alterations that will help us in the development of discussion.

## INTRODUCTION

Lung tumors are very common, both in men and women. They represent the leading cause of cancer death in males and the third most common cause in women. Although the 5-years survival rate has moderately increased in the last years (from 8% to 13%) lung cancer remains associated with a very poor prognosis. [1] The disease is frequently metastatic at diagnosis and therefore treatments are rarely curative. However recently, few but significant steps forward have been made in the treatment of advanced stage non-small cell lung cancer (NSCLC). [2, 3]

In recent years research has focused on molecular characteristics of lung cancer, highlighting the role of specific genes involved in tumor growth. These genes have been shown to be important therapeutic targets, especially in advanced adenocarcinomas. [4] In particular activating *EGFR* mutations were identified in 10-15% of Caucasian and in 40% of Asian patients. Their presence is the most important predictor of good response to *EGFR*-tyrosine-kinase inhibitors (TKIs) therapy. In patients with advanced *EGFR* mutated NSCLC many trials compared chemotherapy and *EGFR*-TKIs in first line setting and showed the clear superiority of the latter. [5] Furthermore after the progression to first and second generation *EGFR*-TKIs (gefitinib, erlotinib and afatinib), the third-generation drugs, have showed very high activity, especially in patients harboring *EGFR* T790M mutation, that is the main cause of resistance to first line TKI therapy. [6, 7]

The rearrangement of *ALK* with *EML4* oncogene on the chromosome 2 short arm is another important therapeutic target. Such aberration activates a specific tyrosine kinase, involved in the processes of survival and cell proliferation and it is found in about 3-7% of lung adenocarcinomas in Caucasian patients. The determination of *EML4-ALK* gene rearrangement is necessary to select patients to be treated with specific *ALK*-TKIs. These drugs have shown to be more active than chemotherapy in all the lines of treatment in which they have been testes, even in patients progressing after one or two different *ALK*-TKIs. [8] These outstanding results underline the need of a thorough molecular characterization of advanced NSCLC, especially in never smokers with non squamous histology. Historically, the coexistence of *EGFR* mutations and *EML4 -ALK* rearrangements have been described as extremely rare, perhaps because of its low prevalence and the sensitivity of diagnostic tools. In contrast recently published case reports and case series suggest that this is not true for all patients [9–11]. In this paper we performed an accurate literature review and we reported two new cases of patients diagnosed with lung adenocarcinoma, exhibiting both *EGFR* mutation and *EML4-ALK* rearrangement. Finally we did a qualitative synthesis of results of the review.

## RESULTS

### Case presentation

#### Case 1

Patient One was a 52-year-old never smoker Caucasian male, diagnosed with stage IV lung adenocarcinoma in May 2012. A CT scan showed lung and bone metastasis at diagnosis. A biopsy of the lung mass revealed the presence of *EGFR* exon 19 deletion and therapy with gefitinib was initiated. In August 2012, after 2 months of gefitinib (250 mg daily), the drug was stopped for liver and skin toxicity. The patient then received two cycles of chemotherapy with cisplatin and gemcitabine and reported progressive disease (PD) as best response. Considering the previous evidence of *EGFR* exon 19 deletion and the discontinuation of gefitinib due to toxicity, we decided to start treatment with erlotinib (150mg daily). Nevertheless lung and bone PD was evident after 4 months. So the patient was treated with pemetrexed and obtained partial response (PR) after 4 cycles and PD on mediastinal nodes after 8 cycles. In December 2013 palliative radiotherapy (RT) (total 30 Gy) on mediastinum and pulmonary hilum was performed. After a new pleural PD the assessment of *EML4-ALK* rearrangement on the original biopsy demonstrated the presence of *EML4-ALK* fusion gene. Crizotinib (500mg daily) was administered from February 2014 until October 2015 when a brain MRI showed the presence of brain metastases. So the patient underwent whole brain radiotherapy (WBRT) and started chemotherapy with vinorelbine. After 3 cycles, complicated by gastrointestinal toxicity, a CT scan showed lung PD and the drug was stopped. In April 2016 the ECOG performance status (PS) of the patient was 2 and he started treatment with ceritinib (750 mg daily) in the context of a clinical trial. Unfortunately, the disease progressed rapidly and the patient died in July 2016.

#### Case 2

Patient Two, a 43-year-old never smoker Caucasian woman, underwent a right lower lobectomy with systematic nodal dissection in April 2012. The initial pathologic diagnosis was lung adenocarcinoma stage IIIa (pT3 pN1 Mx), *KRAS* wild-type, harboring *EGFR* exon 19 deletion and *EML4-ALK* rearrangement. We decided to start adjuvant chemotherapy but in June 2012, before the start of treatment, a brain MRI showed multiple brain metastases and a CT scan demonstrated the presence of lung and nodal disease. So the patient underwent WBRT and started therapy with gefitinib (250 mg daily) which continued until March 2014. She obtained a PR as best response. In March 2014 a new brain MRI demonstrated a slight cerebral PD and the patient was switched to crizotinib (500 mg daily), that was stopped after 4 days because of a G4 gastrointestinal toxicity. Due to the patient's refusal of chemotherapy, she started erlotinib (150 mg daily). She continued therapy until January 2016, obtaining a stabilization of thoracic disease and a slow asymptomatic progression of cerebral disease. In January 2016 the brain MRI showed a significant cerebral PD and a CT scan evidenced lung and pleural PD. The patient was still in good clinical condition (ECOG PS = 0) thus she was enrolled in a clinical trial and received ceritinib (750 mg daily). After 2 cycles a chest/abdomen CT scan and brain MRI showed PR in both lung and brain. At present the patient keeps a good PS and continues ceritinib. The dose was reduced to 450 mg daily due to G2 gastrointestinal toxicity. The disease is stable.

### Descriptive analysis and qualitative synthesis

We identified 98 cases of concomitant *EGFR* mutation and *ALK* rearrangement in NSCLC patients from a literature search. In this paper we will describe a total of 100 cases, including our 2 patients (Table 1). The main patient characteristics are consistent with previous findings about patients with the sole *EGFR* mutation or *EML4-ALK* translocation [52]. There is a prevalence of women (51 female, 31 male, 18 not reported), Asian (53 Asian, 30 Caucasian, 17 not reported) and never smoker patients (58 never smokers, 15 smokers, 27 not reported). We are here considering all patients treated with at least one TKI (*EGFR* or *ALK*-TKIs) and since some patients had more than one treatment with the same class of TKI we are analyzing together all treatments administered for each class (lines of therapy). Fifty one patients received at least one EG*FR*-TKI, with a total of 56 *EGFR*-TKI lines administered. Considering only the 53 *EGFR*-TKI lines evaluable for response, independently from the setting, disease control (CR or PR or SD) was achieved in 37 cases (69.8%) with 23 objective responses reported (43.4%). Thirty-seven patients were treated with at least one *ALK*-TKI (39 evaluable lines of treatment). In these patients disease control was achieved in 31 cases (79.5%) with 20 cases of response (51.3%). Twenty three patients were treated with both *EGFR* and *ALK*-TKIs (27 lines of *EGFR*-TKIs and 27 lines of *ALK*-TKIs administered). Twenty two patients (95.6%) received *EGFR*-TKIs before *ALK*-TKIs. In this subgroup the percentage of disease control and the percentage of objective response with *EGFR*-TKIs were 61.5% (16 of 26 evaluable treatment lines showed CR or PR or SD as best response) and 23.1% (6 of 26 evaluable treatment lines showed CR or PR as best response) respectively whilst with *ALK*-TKIs the percentage of disease control and the percentage of objective response were 73.1% (19 of 26 evaluable treatment lines) and 42.3% (11 of 26 evaluable treatment lines) respectively. (Table 2).

**Table 1:** Clinical and biological characteristics of patients with both *EML4-ALK* rearrangement and *EGFR* mutation

Author	Pts characteristics	Biomolecular characterization	*EGFR*-TKI treatment	*ALK*-TKI treatment	Other treatments
Zhao N et al [9]	1 pts. F,48 Y, NS, Asiatic	-*EGFR* exon 21 (L861Q) mutation -*EML4-ALK* variant NR	-first line erlotinib. BR: SD (PFS 5,3 mts)	-third line crizotinib. BR:SD (PFS 3,5 mts) -fourth line brigatinib. BR:PR	-second line nedaplatin + pemetrexed. BR: PD (3 cycles) -fifth line axitinib. BR: PD
Sweis RF et al [10]	4 pts. 2M 2F, median age 53 Y, 2 CS 2 NS, Asiatic	-*EGFR*: 1 pts exon 19 deletion, 1pts exon 21 (L861Q) mutation, 1pts exon 21 (L858R) mutation, 1 pts exon 23 polymorphism -*EML4-ALK* variant NR	-pts N 1 Manteinance therapy erlotinib + bevacizumab. BR: PR (PFS 12 mts) -pts N 3 first line erlotinib. BR: PD (PFS 2 mts) -pts N 4 third line erlotinib. BR: PD (PFS 2 mts)	-pts N 1 third line crizotinib. BR: SD (PFS 9 mts) -pts N2 third line crizotinib. BR: PD (PFS 1 mts) -pts N 3 second line crizotinib. BR: PD (PFS 1 mts) -pts N 4 first line crizotinib. BR:PD	-pts N 1 first line carboplatin + paclitaxel + bevacizumab. BR: PR (6 cycles) -pts N 1 second line: pemetrexed. BR: PD (PFS 3 mts) -pts N2 first line carboplatin + pemetrexed. BR: PR (6 cycles) -pts N2 second-line docetaxel. BR: PD -pts N 4 second-line carboplatin + pemetrexed. BR: PR (4 cycles)
Xu CW et al [11]	1 pts. F, 71 Y, NS, Asiatic	-*EGFR* exon 19 deletion -*EML4-ALK* variant 1	-first line gefitinib. BR: PR	NT	NT
Zhang X et al [20]	1 pts. F, NS, Asiatic	-*EGFR* exon 19 deletion -*EML4-ALK* variant 3 b	NR	NT	NR
Koivunen JP et al [21]	1 pts. NR clinical data	-*EGFR* exon 19 deletion -*EML4-ALK* variant NR	NT	NT	NT
Kuo YW [22]	1 pts. F,72 Y, NS, Asiatic	-*EGFR* exon 19 deletion -*EML4-ALK* variant 1	-first line gefitinib. BR: PR (PFS 7 mts)	NT	NR
Sasaki T et al [23]	3 pts. NR clinical data	-*EGFR*: 1 pts exon 19 deletion, 1pts exon 21 (L858R) mutation, 1 pts mutation A767_V769dupASV -*EML4-ALK* variant NR	-2 pts treated with erlotinib. BR: 2 pts PR (9 mts, 5 mts PFS respectively)	NT	NR
Tiseo M et al [24]	1 pts. M,48 Y, NS, Caucasian	-*EGFR* exon 19 deletion -*EML4-ALK* variant 1	-second line erlotinib. BR: PD (PFS 2 mts)	NT	-first line cisplatin + gemcitabine. BR: PR (4 cycles).
Popat S et al [25]	1 pts. F, 65 Y, NS, Caucasian	-*EGFR* exon 19 deletion -*EML4-ALK* variant NR	-first line erlotinib. BR: CR (PFS 25 mts)	NT	-adjuvant carboplatin + vinorelbine (4 cycles)
Tanaka H et al [27]	1 pts. M, 39 Y, LS, Asiatic	-*EGFR* exon 21 (L858R) mutation -*EML4-ALK* variant 3B	-third line erlotinib. BR: PD (PFS 1 mts)	NT	-first line cisplatin + docetaxel. BR: PD (3 cycles) -second line pemetrexed. BR:PR (15 cycles)-fourth line NR chemotherapy. BR: PD
Lee JK et al [28]	4pts. 1 pts M,73 Y, LS, Asiatic. 3 pts NR clinical data	-*EGFR* 2 pts exon 19 deletion, 1pts exon 21 (L858R) mutation, 1 pts exon 21 (L718P) mutation -*EML4-ALK* variant NR	-1 pts first line gefitinib. BR: PD (PFS 1 mts)	-1 pts second line crizotinib. BR: PR (PFS 9 mts)	NT
Pilotto et al [29]	1 pts. F,78Y, NS, Caucasian	-*EGFR* exon 21 (L861Q) mutation -*EML4-ALK* increased GCG	-first line gefitinib. BR: PD (PFS 2 mts)	-second line crizotinib. BR: SD (PFS 4 mts)	NT
Miyanaga et al [30]	1 pts. F, 55 Y, NS, Asiatic	-*EGFR* exon 19 deletion -*EML4-ALK* variant 2	-second line gefitinib. BR:PD (PFS 2 mts) -third line erlotinib. BR: PD (PFS 3 mts)	-fifth line NR ALK TKI. BR: SD (PFS 4 mts)	-first line cisplatin + pemetrexed. BR: SD (4 cycles) -fourth line docetaxel. BR: PD (2 cycles)
Chen X et al [31]	1 pts. M,56 Y, HS, Asiatic	-*EGFR* exon 19 deletion -*EML4-ALK* variant NR	-second line erlotinib. BR:SD	-third line crizotinib. BR:CR	-first line cisplatin + gemcitabine (1 cycle interrupted for toxicity)
Santelmo C et al [32]	1 pts. F,52 Y, HS, Caucasian	-*EGFR* exon 19 deletion -*EML4-ALK* variant NR	-neoadjuvant gefitinib. BR:PR	NT	NT
Chiari R et al [33]	1 pts. F,67 Y, NS Caucasian	-*EGFR* exon 21 (L858R) mutation -*EML4-ALK* variant NR	-third line erlotinib/afatinib. BR:SD	-sixth line crizotinib. BR:PR	-first line cisplatin + gemcitabine. BR: SD (6 cycles) -second line carboplatin + pemetrexed. BR: SD (8 cycles) -fourth line docetaxel. BR: SD (10 cycles) -fifth line pemetrexed. BR: SD (6 cycles)
Yang JJ et al [34]	13 pts. 8 F 5 M, 12 NS 1 LS, 59 Y (median), Asiatic	-*EGFR*: 7 pts exon 19 deletion, 4 pts exon 21 (L858R) mutation, 1pts exon 20 mutation, 1pts K757 R mutation -*EML4-ALK* variant: 5 pts V1, 2 pts V3a/V3b, 1 pts V4b, 1 pts V5, 1 pts V6b, 3 pts NA	-10 pts receiving first-line *EGFR*-TKI. BR: 8 PR (4 pts, 3 pts, and 1pts treated with erlotinib, gefitinib, and afatinib respectively), 1 SD (afatinib); and 1 PD (erlotinib). ORR: 80% (8/10 pts). Median PFS: 11.2 mts	-1 pts received first line crizotinib. BR:PR (PFS 15 mts) (*NR previous EGFR TKI treatment*)-3 pts received second line crizotinib. BR: 1 pts PR, 1 pts PD, 1 pts SD *(all pts previously treated with EGFR TKI. BR: 1 pts PD, 1 pts PR, 1 pts PR)*	NR
Baldi et al [35]	1 pts. M, 68 Y, NS, Caucasian	-*EGFR* exon 21 (L858R) mutation -*EML4-ALK* variant NR	-second line erlotinib.BR:SD (PFS 3 years)	-third line crizotinib. BR: PR	-first-line cisplatin + pemetrexed. After 4 cycles BR:SD. Maintenance pemetrexed/ placebo. BR:SD (15 cycles)
Jurgens et al [36]	1 pts. M, 69 Y, LS, Caucasian	-*EGFR* exon 21 (861) mutation -*EML4-ALK* variant NR	-first line gefitinib. BR:PD	NT	-second line carboplatin + pemetrexed + bevacizumab. BR: PR
Cabillic F et al [37]	8 pts. 5 M 3 F, median age 65 Y, Smoke NR, Caucasian	-*EGFR*: 3 pts exon 19 deletion, 4 pts exon 21 (L858R) mutation, 1 pts L858R + T790 M exon 21 mutation -*EML4-ALK* variant NR	-2 pts first line gefitinib. BR:NR	-1 pts second line crizotinib. BR: PR *(NR previous EGFR-TKI treatment)*	NR
Wang J et al [38]	1 pts. Asiatic. Other clinical data NR	-*EGFR* exon21 (L858R) mutation -*EML4-ALK* variant NR	NR	NR	NR
Kim TJ et al [39]	5 pts. 3 M 2 F, median age 62 Y, 4 NS 1 HS, Asiatic	-*EGFR*: 3 pts exon 19 deletion, 2 pts exon 21 (L858R) mutation -*EML4-ALK* variant NR	NR	NR	NR
Won JK et al [40]	14 pts *(1 pts previously reported by Lee JK et al)*. 6 M 8 F, median age 55 Y, 12 NS 2 LS, Asiatic	-*EGFR*: 9 pts exon 19 deletion, 2 pts exon 19 L747P, 1 pts exon 21 L861Q, 1 pts exon 21 L858R, 1 pts exon 21 E868K -*EML4-ALK* variant NR	-3 pts treated with gefitinib. BR: 2 pts PD, 1 pts SD (PFS 6 mts)	-6 pts treated with crizotinib. BR: 5 pts PR 1 pts SD *(only 1 pts previously treated with gefitinib PD→PR)* -2 pts treated with ceritinib. BR: 2pts PR *(NR previous EGFR-TKI treatment*)	NR
Inamura et al [41]	1 pts. F, 55 Y, NS, Asiatic	-*EGFR* exon19 deletion -*EML4-ALK* variant 1	-second line gefitinib. BR:PR (PFS 31 mts)-21 th line alectinib + gefitinib. BR:SD (PFS 5 mts)-22 th line third generation of *EGFR*-TKIs. BR:PR	-19 th line crizotinib. BR:SD -20 th line alectinib. BR:PD -21 th line alectinib + gefitinib. BR:SD	-over 20 lines of treatment. Cytotoxic chemotherapy was generally less effective
Caliez et al [42]	2 pts. 2 F, 64 and 45 Y, 1 NS,1 LS, Caucasian	-*EGFR* mutation NR -*EML4-ALK* variant NR	-Pts N1 second line erlotinib. BR:SD ( PFS 14 mts) -Pts N 2 second line afatinib. BR:SD (PFS 8 mts)	-Pts N1 forth line crizotinib. BR:PR (PFS 24 mts) -Pts N2 third line crizotinib. BR:PD (PFS 1 mts)	-Pts N1 first line cisplatin + gemcitabine, followed by 17 months of gemcitabine. BR:PR-Pts N1 third line paclitaxel (PFS:6 mts)-Pts N2 first line cisplatin + gemcitabine (PFS 8 mts)
Galetta et al [43]	1 pts. F, 76 Y, NS, Caucasian	-*EGFR* exon 19 deletion -*EML4-ALK* variant NR	-second line gefitinib. BR:PR (PFS 6 mts)	-third line crizotinib. BR:PD	-first line cisplatin + pemetrexed. BR:PR
Fan T [44] et al	1 pts. F, 63 Y, NS, Asiatic	-*EGFR* exon19 deletion -*EML4-ALK* variant NR	NT	NT	-first line carboplatin + pemetrexed. BR:NR
Zhou J et al [45]	1 pts. F, 47 Y, NS, Asiatic	-*EGFR* exon 19 deletion -*EML4-ALK* variant NR	-second line gefitinib. BR:PD (PFS 2 mts)	NT	-first line cisplatin + gemcitabine. BR:NR
Rossing HH et al [46]	1 pts. M, 61 Y, NS, Caucasian	-*EML4-ALK* variant NR-acquired *EGFR* exon 21 (L862R)mutation -acquired *KRAS* codon 13 (c.38G > A, p.G13D) mutation	NT	-second line crizotinib. BR:PR (PFS 8 mts)	-first line carboplatin-vinorelbine -bevacizumab. BR:PR-third-line pemetrexed. BR:PD
Lee T et al [48]	6 pts. 1 M 5 F, median age 62 Y, 5 NS 1 LS, Asiatic	-*EGFR*: 2 pts exon 18 G719X, 1 pts exon 21 L858R, 1 pts both exon 18 G719X and exon 21 L858R, 1 pts exon 19 deletion, 1 pts exon 20 R803W -*EML4-ALK* variant NR	-2 pts treated with gefitinib. BR:1 pts PR 1 pts SD -1 pts treated with erlotinib BR:PD	3 pts treated with crizotinib.BR: 1 pts PR, 1pts SD, 1 pts NR. (*Only 1 pts previously treated with gefitinib SD→PR)*	NR
Ulivi et al [49]	6 pts.1 M 5 F, median age 60 Y,3 NS 2 FS 1CS, Caucasian	-*EGFR*: 5 pts exon 19 deletion, 1 pts exon 21 (L858R) mutation. -*EML4-ALK* variant NR	-5 pts first line gefitinib.BR: 1 pts CR,2 pts PR,2 pts PD -1 pts first line erlotinib. BR:PR	-1 pts second line crizotinib. BR:NR. *(NR previous EGFR TKI treatment)*	NR
Sahnane N et al [50]	3 pts. 1 M 2 F, 51-67-74 Y, 2 NS 1 NR, Caucasian	-*EGFR*: 1 pts exon 19 deletion, 1 pts exon 21 (L858R) mutation, 1 pts exon 18 G719A mutation -*EML4-ALK* variant NR	-2 pts first line erlotinib. BR: 1 pts PD,1 pts SD (PFS 8 mts)	-1 pts second line crizotinib. BR:SD*(previously treated with erlotinib.BR:SD)* -1 pts third line crizotinib. BR: PR *(previously treated with erlotinib.BR:PD)*	-1 pts second line carboplatin + gemcitabine. BR:PD ( 3 cycles)
Guibert N et al [52]	10 pts. Other clinical data NR	-EGFR mutation NR -*EML4-ALK* variant NR	NR	-1 pts treated with crizotinib. BR:SD	NR
Our cases	2 pts. 1 M 1 F, 52-43 Y, 2 NS, Caucasian	-*EGFR*: 2 pts exon 19 deletion -*EML4-ALK* variant NR	-Pts N1first line geftinib for 2 months (stopped for toxicity). BR:NE -Pts N1 third line erlotinib. BR:PD -Pts N2 first line geftinib. BR:PD -Pts N2 third line erlotinib. BR:SD	-Pts N1 fifth line crizotinib. BR:PR (PFS 24 mts) -Pts N1 7^th^ line ceritinib. BR:PD -Pts N2 second line crizotinib (stopped for toxicity). BR:NE -Pts N2 forth line ceritinib. BR: PR	-Pts N1second line cisplatin + gemcitabine. BR:PD -Pts N1 forth line pemetrexed.BR:PR -Pts N1 sixth line vinorelbine. BR:PD

*EML4-ALK* = echinoderm microtubule associated protein like 4 -anaplastic lymphoma kinase, *EGFR* = epidermal growth factor receptor, pts = patients, TKI = tyrosine-kinase inhibitor, F = female, Y = years, mts = months, NS = never smokers, NR = not reported, BR = best response, N = number, SD = stable disease, PFS = progression free survival, PR = partial response, PD = progressive disease, M = male, CS = current smokers, NT = not treated, HS = high smokers, CR = complete response, LH = light smokers, FS = former smokers, NE = not evaluable.

**Table 2:** Qualitative synthesis of reported best responses

Treatment	N Pts	N Lines#	N CR+PR	N SD	N PD	CR+PR (%)	DC (%)
*EGFR*-TKIs	51	53	23	14	16	43,4	69.8
*ALK*-TKIs	37	39	20	11	8	51.3	79.5
*EGFR* + *ALK*-TKIs	23*	-26 *EGFR*-TKI lines -26 *ALK*-TKI lines	-6 with *EGFR*-TKIs -11 with *ALK*-TKIs	-10 with *EGFR*-TKIs -8 with *ALK*-TKIs	-10 with *EGFR*-TKIs -7 with *ALK*-TKIs	-23.1 with *EGFR*-TKIs -42.3 with *ALK*-TKIs	-61.5 with *EGFR*-TKIs -73.1 with *ALK*-TKIs

N = number, Pts = patients, CR = complete response, PR = partial response, SD = stable disease, PD = progressive disease, NE = not evaluable, DC = disease control, *EGFR*-TKI = epidermal growth factor receptor tyrosine-kinase inhibitor, *ALK*-TKI = anaplastic lymphoma kinase receptor tyrosine-kinase inhibitor.

* Twenty two patients (95.6%) received first *EGFR*-TKI before the first *ALK*-TKI.

# Evaluable for response.

## DISCUSSION

In recent years the study of molecular characteristics of lung cancer has underlined the pathogenic and therapeutic importance of specific genes involved in tumor growth. The most significant discovery was undoubtedly the role as therapeutic target of *EGFR* and *ALK* in the non squamous histology, but many other genes involved in the cell proliferation process have been investigated. The oncogene *KRAS* is mutated in 20-30% of lung adenocarcinomas, particularly in heavy smokers and in the Caucasian population. However its role is still controversial both as a prognostic and predictive factor. Other molecular alterations have already demonstrated a promising therapeutic impact, especially in lung adenocarcinomas. They are represented by the *ROS1* and *RET* gene rearrangements (about 1-2% of lung adenocarcinomas) and by the activating mutations of *BRAF* (V600E and others) and *HER2*. Also the FGFR1 and *PDGFR* amplification and the mutations of *PI3KCA*, *PTEN* and *DDR2* could have future therapeutic implications, especially in squamous cell lung cancer. [4, 12]. Anyhow at present only the discovery of *EGFR* and *ALK* molecular alterations has radically changed the clinical history of two specific subgroup of patients (*EGFR* mutated and *EML4-ALK* rearranged patients). [13, 14] In the first years after its discovery the presence of *ALK* rearrangement has been described as mutually exclusive with the presence of *EGFR* mutations [15–20]. Some authors reported that *ALK* rearrangements were more common in patients with poorly differentiated adenocarcinoma whilst *EGFR* mutations were typical of well-differentiated cancers [15]. In the same way the coexistence of *KRAS* mutations with the other two alterations was described as virtually impossible. Thus the initial suggested algorithms for bio-molecular characterization of non squamous NSCLC proposed testing the histological samples for *KRAS* mutations followed by *EGFR* test only if *KRAS* was wild type. *ALK* testing was to be performed only if no alterations were found in previous tests [16–20].

However a higher number of subsequent observations demonstrated that the concomitant presence of *EML4-ALK* rearrangements and *EGFR* mutations is rare but not irrelevant [9–11, 21–45] (Table 1), and also the coexistence with *KRAS* and other mutations is possible [46–52]. So at present this diagnostic algorithm cannot be considered the gold standard.

In regards to patients with both *EML4-ALK* rearrangements and *EGFR* mutations, literature data are conflicting. Yang et al [34] and Ulivi et al [49] reported a percentage of concurrent alterations of 1.3% and 1.6% respectively, whilst Lee et al described only 6 cases of concomitant alterations on 6637 NSCLC cases [48]. In some cases the presence of *EGFR* mutations is described as a resistance mechanism occurring in patients with *EML4-ALK* translocations treated with *ALK*-TKIs [46, 51]. At the same time the development of *EML4-ALK* rearrangements could be an acquired resistance mechanism after *EGFR*-TKIs treatment, although the second event is considered more rare [23, 41]. Other authors reported that both genetic alterations could be present from the beginning of tumor proliferation [23, 34, 35, 39]. The different cellular clones represent the expression of the heterogeneity of tumors [39]. Moreover these two bio-molecular alterations may coexist in the same tumor cell [35]. In our opinion all mechanisms are possible and can probably occur in the same patient during the course of the disease.

Another hot topic is the variable response reported to both *ALK*-TKIs and *EGFR*-TKIs in the different published cases. Some authors hypothesized that the coexistence of two alterations could be predictive of poor response in patients treated with TKIs [24, 43, 46]. These data however, were not confirmed by other authors, that reported a good sensitivity to these treatments [22, 25, 33]. Furthermore some papers showed a better outcome only in patients treated with first line *ALK*-TKIs [34, 40, 48] whilst an unsatisfactory response was reported with first line *EGFR*-TKIs [30, 31, 35]. Again these results are not confirmed by other experiences [22, 25, 33, 52].

Despite the increasing number of reported cases of coexistent alterations, these are still not frequent enough to allow the authors to define specific guidelines and at present it is not possible to establish the best sequence of treatments. The two cases reported here are an example of this ambiguous context. Both patients had concomitant *EGFR* mutation and *ALK* rearrangement *ab initio*. They were both treated upfront with an *EGFR*-TKI (gefitinib) followed by another drug of the same family (erlotinib). The first patient did not respond to either treatments, while in the second case we obtained a PR with gefitinib and a long SD with erlotinib. Both patients were also treated with two *ALK*-TKIs (crizotinib and ceritinib). The first patient achieved a long SD after crizotinib but did not respond to ceritinib. In the second case the response to crizotinib was not evaluable because it was withdrawn due to an important toxicity, but the patient reported an outstanding response with ceritinib. Thus also in our experience, data are very dishomogeneous and do not allow us to draw any conclusions. Nevertheless we can give some suggestions on the basis of the qualitative data synthesis.

Considering literature data and the great efficacy of both *ALK* and *EGFR*-TKIs, it is strongly advised that *EGFR* and *ALK* tests are performed *ab initio* in all advanced non squamous NSCLC.

Although there is no literature consensus, and our analysis is only a descriptive non statistical data synthesis, *ALK*-TKIs seem to be slightly more effective than *EGFR*-TKIs in patients with both alterations (Table 2). Disease control or disease response are reported as best response in 69.8% and 43.4% versus 79.5% and 51.3% of reviewed cases treated with *EGFR* and *ALK*-TKIs respectively. Twenty three patients received both *EGFR* and *ALK*-TKIs. In the great majority of cases (22 patients) *EGFR*-TKIs were administered before *ALK*-TKIs. Despite this fact, in this subgroup of patients, disease control was achieved in 61.5% versus 73.1% and disease response was seen in 23.1% versus 42.3% of cases treated with *EGFR* versus *ALK*-TKIs respectively. Furthermore also the role of chemotherapy in these patients is still debated [52]. Obviously, the very limited number of evaluable patients does not allow us to draw definitive conclusions but in our opinion, *ALK*-TKIs treatment could be the preferred first line approach, if there aren't any other data that could guide the therapeutic choice.

*EGFR* mutations, *ALK* translocations and many other bio-molecular alterations [53] may appear in the course of the disease as acquired resistance mechanisms after *EGFR* and *ALK*-TKIs treatment. Thus, if technically feasible, it is crucial to re-biopsy all patients with PD in order to detect all possible new alterations and design therapy on the basis of the new bio-molecular status.

Patients with *EGFR* mutations and *EML4-ALK* translocations have a higher incidence of brain metastases [54]. These are also present in our two cases. To date WBRT and Stereotactic Radiosurgery (SRS) represent the most used treatments in patients with intracranial involvement [55]. Nevertheless *EGFR* and *ALK*-TKIs, especially the new-generation drugs, have shown relevant intracranial activity [56–58]. Therefore the TKIs role and their correct place within the therapeutic strategy are still debated, especially for patients with brain metastasis and both alterations.

Since the two alterations may coexist *ab initio*, another very interesting future perspective could be to test the safety and the potential efficacy of a double inhibition of both *ALK* and *EGFR*. Designing clinical trials specific for these patients could be very fascinating and could answer many open questions. However, due the rarity of this condition, it would be difficult to recruit the right number of patients.

In conclusion, an accurate bio-molecular characterization is crucial to drive the therapeutic strategy of NSCLC with multiple driver alterations. Perhaps the allelic alteration's fraction in NGS or the liquid biopsies could be useful in defining the best therapeutic choice [52]. All available clinical data should be shared and analyzed in order to increase our experiences and correctly manage these patients.

## MATERIALS AND METHODS

We performed a literature review on reported cases and case series of concomitant *EML4-ALK* rearrangement and *EGFR* mutation in patients with NSCLC. We collected all available clinical data and investigated treatment strategies used in the management of this rare group of tumors. We searched the digital databases using these keywords: “Non-Small Cell Lung Cancer”, “*EML4-ALK* rearrangements”**, “***EGFR* mutations”, variously associated with “concomitant”, “coexistence”, “coexistent”, “concurrent”, “both”, “same tumor”, “same cancer”, “same neoplasm”. We did not restrict the search to English language but we included in the analysis only the publications for which an English abstract was available. We focused our attention mainly on the drugs used and treatment efficacy. We read the full text of selected papers (abstracts were analyzed when the English version of the article was not present). The reference list of selected papers was also used in the selection process.

**Figure 1:**
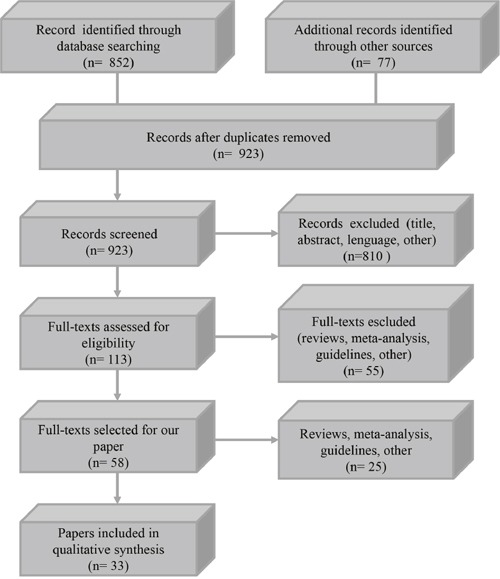
Flow diagram of the study selection process

We examined 923 papers, 865 works were excluded based on title and abstract content while, the remaining 58 papers were included in our review. Thirty three studies, were selected for qualitative synthesis (Table 1), (Figure 1).

CP, DS, MV, MG, LB, NZ: acquisition of data; analysis and interpretation of data; critical revision of the manuscript; study supervision.

## References

[1] Francisci S, Minicozzi P, Pierannunzio D, Ardanaz E, Eberle A, Grimsrud TK, Knijn A, Pastorino U, SalmerónD Trama A, M; Sant, EUROCARE-5 Working Group (2015). Survival patterns in lung and pleural cancer in Europe 1999-2007: results from the EUROCARE-5 study. Eur J Cancer.

[2] Rossi A, Torri V, Garassino MC, Porcu L, Galetta D (2014). The impact of personalized medicine on survival: comparisons of results in metastatic breast, colorectal and non-small-cell lung cancers. Cancer Treat Rev.

[3] Reck M, Popat S, Reinmuth N, De Ruysscher D, Kerr KM, Peters S, ESMO Guidelines Working Group (2014). Metastatic non small- cell lung cancer (NSCLC): ESMO Clinical Practice Guidelines for diagnosis, treatment and follow-up. Ann Oncol.

[4] Dearden S, Stevens J, Wu YL, Blowers D (2013). Mutation incidence and coincidence in non small-cell lung cancer: meta-analyses by ethnicity and histology (mutMap). Ann Oncol.

[5] Lee CK, Brown C, Gralla RJ, Hirsh V, Thongprasert S, Tsai CM, Tan EH, Ho JC, Chu da T, Zaatar A, Osorio Sanchez JA, Vu VV, Au JS (2013). Impact of EGFR inhibitor in non-small cell lung cancer on progression-free and overall survival: a meta-analysis. J Natl Cancer Inst.

[6] Jänne PA, Yang JC, Kim DW, Planchard D, Ohe Y, Ramalingam SS, Ahn MJ, Kim SW, Su WC, Horn L, Haggstrom D, Felip E, Kim JH (2015). AZD9291 in EGFR inhibitor- resistant non-small-cell lung cancer. N Engl J Med.

[7] Sequist LV, Soria JC, Goldman JW, Wakelee HA, Gadgeel SM, Varga A, Papadimitrakopoulou V, Solomon BJ, Oxnard GR, Dziadziuszko R, Aisner DL, Doebele RC, Galasso C (2015). Rociletinib in EGFR-mutated non-small-cell lung cancer. N Engl J Med.

[8] Passaro A, Lazzari C, Karachaliou N, Spitaleri G, Pochesci A, Catania C, Rosell R, de Marinis F (2016). Personalized treatment in advanced ALK-positive non-small cell lung cancer: from bench to clinical practice. Onco Targets Ther.

[9] Zhao N, Zheng SY, Yang JJ, Zhang XC, Xie Z, Xie B, Su J, Chen ZH, Chen SL, Zhang N, Lou NN, Dong S, Wu YL (2015). Lung adenocarcinoma harboring concomitant EGFR mutation and EML4-ALK fusion that benefits from three kinds of tyrosine kinase inhibitors: a case report and literature review. Clin Lung Cancer.

[10] Sweis RF, Thomas S, Bank B, Fishkin P, Mooney C, Salgia R (2016). Concurrent EGFR mutation and ALK translocation in non-small cell lung cancer. Cureus.

[11] Xu CW, Cai XY, Shao Y, Li Y, Shi MW, Zhang LY, Wang L, Zhang YP, Wang LP, Tian YW (2015). A case of lung adenocarcinoma with a concurrent EGFR mutation and ALK rearrangement: a case report and literature review. Mol Med Rep.

[12] Cancer Genome Atlas Research Network (2012). Comprehensive genomic characterization of squamous cell lung cancers. Nature.

[13] Sequist LV, Joshi VA, Jänne PA, Muzikansky A, Fidias P, Meyerson M, Haber DA, Kucherlapati R, Johnson BE, Lynch TJ (2007). Response to treatment and survival of patients with non-small cell lung cancer undergoing somatic EGFR mutation testing. Oncologist.

[14] Kwak EL, Bang YJ, Camidge DR, Shaw AT, Solomon B, Maki RG, Ou SH, Dezube BJ, Jänne PA, Costa DB, Varella-Garcia M, Kim WH, Lynch TJ (2010). Anaplastic lymphoma kinase inhibition in non-small-cell lung cancer. N Engl J Med.

[15] Xu L, Lei J, Wang QZ, Li J, Wu L (2015). Clinical characteristics of patients with non-small cell lung cancers harboring anaplastic lymphoma kinase rearrangements and primary lung adenocarcinoma harboring epidermal growth factor receptor mutations. Genet Mol Res.

[16] Lindeman NI, Cagle PT, Beasley MB, Chitale DA, Dacic S, Giaccone G, Jenkins RB, Kwiatkowski DJ, Saldivar JS, Squire J, Thunnissen E, Ladanyi M, College of American Pathologists International Association for the Study of Lung Cancer and Association for Molecular Pathology (2013). Molecular testing guideline for selection of lung cancer patients for EGFR and ALK tyrosine kinase inhibitors: guideline from the College of American Molecular Pathologists, International Association for the Study of Lung Cancer, and Association for Molecular Pathology. J Mol Diagn.

[17] Wang Y, Wang S, Xu S, Qu J, Liu B (2014). Clinicopathologic features of patients with non-small cell lung cancer harboring the EML4-ALK fusion gene: a meta-analysis. PLoS One.

[18] Hsu SC, Hung TH, Wang CW, Ng KF, Chen TC (2015). Anaplastic lymphoma kinase translocation is correlated with anaplastic lymphoma kinase expression and mutually exclusive with epidermal growth factor receptor mutation in Taiwanese non-small cell lung cancer. Pathol Int.

[19] Gainor JF, Varghese AM, Ou SH, Kabraji S, Awad MM, Katayama R, Pawlak A, Mino-Kenudson M, Yeap BY, Riely GJ, Iafrate AJ, Arcila ME, Ladanyi M (2013). ALK rearrangements are mutually exclusive with mutations in EGFR or KRAS: an analysis of 1,683 patients with non-small cell lung cancer. Clin Cancer Res.

[20] Zhang X, Zhang S, Yang X, Yang J, Zhou Q, Yin L, An S, Lin J, Chen S, Xie Z, Zhu M, Zhang X, Wu YL (2010). Fusion of EML4 and ALK is associated with development of lung adenocarcinomas lacking EGFR and KRAS mutations and is correlated with ALK expression. Mol Cancer.

[21] Koivunen JP, Mermel C, Zejnullahu K, Murphy C, Lifshits E, Holmes AJ, Choi HG, Kim J, Chiang D, Thomas R, Lee J, Richards WG, Sugarbaker DJ (2008). EML4-ALK fusion gene and efficacy of an ALK kinase inhibitor in lung cancer. Clin Cancer Res.

[22] Kuo YW, Wu SG, Ho CC, Shih JY (2010). Good response to gefitinib in lung adenocarcinoma harboring coexisting EML4-ALK fusion gene and EGFR mutation. J Thorac Oncol.

[23] Sasaki T, Koivunen J, Ogino A, Yanagita M, Nikiforow S, Zheng W, Lathan C, Marcoux JP, Du J, Okuda K, Capelletti M, Shimamura T, Ercan D (2011). A novel ALK secondary mutation and EGFR signaling cause resistance to ALK kinase inhibitors. Cancer Res.

[24] Tiseo M, Gelsomino F, Boggiani D, Bortesi B, Bartolotti M, Bozzetti C, Sammarelli G, Thai E, Ardizzoni A (2011). EGFR and EML4-ALK gene mutations in NSCLC: a case report of erlotinib-resistant patient with both concomitant mutations. Lung Cancer.

[25] Popat S, Vieira de Araújo A, Min T, Swansbury J, Dainton M, Wotherspoon A, Lim E, Nicholson AG, O'Brien ME (2011). Lung adenocarcinoma with concurrent exon 19 EGFR mutation and ALK rearrangement responding to erlotinib. J Thorac Oncol.

[26] Zeng Z, Wu Y (2011). [Research progress in non-small cell lung cancer with concomitant EML4-ALK fusion gene and EGFR gene mutation]. [Article in Chinese]. Zhongguo Fei Ai Za Zhi.

[27] Tanaka H, Hayashi A, Morimoto T, Taima K, Tanaka Y, Shimada M, Kurose A, Takanashi S, Okumura K (2012). A case of lung adenocarcinoma harboring EGFR mutation and EML4-ALK fusion gene. BMC Cancer.

[28] Lee JK, Kim TM, Koh Y, Lee SH, Kim DW, Jeon YK, Chung DH, Yang SC, Kim YT, Kim YW, Heo DS, Bang YJ (2012). Differential sensitivities to tyrosine kinase inhibitors in NSCLC harboring EGFR mutation and ALK translocation. Lung Cancer.

[29] Pilotto S, Bria E, Peretti U, Massari F, Garassino M, Pelosi G, Tortora G (2013). Lung adenocarcinoma patient refractory to gefitinib and responsive to crizotinib, with concurrent rare mutation of the epidermal growth factor receptor (L861Q) and increased ALK/MET/ROS1 gene copy number. J Thorac Oncol.

[30] Miyanaga A, Shimizu K, Noro R, Seike M, Kitamura K, Kosaihira S, Minegishi Y, Shukuya T, Yoshimura A, Kawamoto M, Tsuchiya S, Hagiwara K, Soda M (2013). Activity of EGFR-tyrosine kinase and ALK inhibitors for EML4–ALK-rearranged non–small–cell lung cancer harbored coexisting EGFR mutation. BMC Cancer.

[31] Chen X, Zhang J, Hu Q, Li X, Zhou C (2013). A case of lung adenocarcinoma harboring exon 19 EGFR deletion and EML4-ALK fusion gene. Lung Cancer.

[32] Santelmo C, Ravaioli A, Barzotti E, Papi M, Poggi B, Drudi F, Mangianti M, Salvi M, Crinò L (2013). Coexistence of EGFR mutation and ALK translocation in NSCLC: literature review and case report of response to gefitinib. Lung Cancer.

[33] Chiari R, Duranti S, Ludovini V, Bellezza G, Pireddu A, Minotti V, Bennati C, Crinò L (2014). Long-term response to gefitinib and crizotinib in lung adenocarcinoma harboring both epidermal growth factor receptor mutation and EML4-ALK fusion gene. J Clin Oncol.

[34] Yang JJ, Zhang XC, Su J, Xu CR, Zhou Q, Tian HX, Xie Z, Chen HJ, Huang YS, Jiang BY, Wang Z, Wang BC, Yang XN (2014). Lung cancers with concomitant EGFR mutations and ALK rearrangements: diverse responses to EGFR-TKI and crizotinib in relation to diverse receptors phosphorylation. Clin Cancer Res.

[35] Baldi L, Mengoli MC, Bisagni A, Banzi MC, Boni C, Rossi G (2014). Concomitant EGFR mutation and ALK rearrangement in lung adenocarcinoma is more frequent than expected: report of a case and review of the literature with demonstration of genes alteration into the same tumor cells. Lung Cancer.

[36] Jürgens J, Engel-Riedel W, Prickartz A, Ludwig C, Schildgen O, Tillmann RL, Stoelben E, Brockmann M, Schildgen V (2014). Combined point mutation in KRAS or EGFR genes and EML4-ALK translocationin lung cancer patients. Future Oncol.

[37] Cabillic F, Gros A, Dugay F, Begueret H, Mesturoux L, Chiforeanu DC, Dufrenot L, Jauffret V, Dachary D, Corre R, Lespagnol A, Soler G, Dagher J (2014). Parallel FISH and immunohistochemical studies of ALK status in3244 non-small-cell lung cancers reveal major discordances. J Thorac Oncol.

[38] Wang J, Dong Y, Cai Y, Zhou L, Wu S, Liu G, Su D, Li X, Qin N, Nong J, Jia H, Zhang Q, Mu J (2014). Clinicopathologic characteristics of ALK rearrangements in primary lung adenocarcinoma with identified EGFR and KRAS status. J Cancer Res Clin Oncol.

[39] Kim TJ, Park CK, Yeo CD, Park K, Rhee CK, Kim J, Kim SJ, Lee SH, Lee KY, Yoon HK (2014). Simultaneous diagnostic platform of genotyping EGFR, KRAS, and ALK in 510 Korean patients with non-small-cell lung cancer highlights significantly higher ALK rearrangement rate in advanced stage. J Surg Oncol.

[40] Won JK, Keam B, Koh J, Cho HJ, Jeon YK, Kim TM, Lee SH, Lee DS, Kim DW, Chung DH (2015). Concomitant ALK translocation and EGFR mutation in lung cancer: a comparison of direct sequencing and sensitive assays and the impact on responsiveness to tyrosine kinase inhibitor. Ann Oncol.

[41] Imamura F, Inoue T, Kimura M, Nishino K, Kumagai T (2016). A long-term survivor of non-small-cell lung cancer harboring concomitant EGFR mutation and ALK translocation. Respir Med Case Rep.

[42] Caliez J, Monnet I, Pujals A, Rousseau-Bussac G, Jabot L, Boudjemaa A, Leroy K, Chouaid C (2017). [Lung adenocarcinoma with concomitant EGFR mutation and ALK rearrangement]. [Article in French]. Rev Mal Respir.

[43] Galetta D, Catino A, Misino A (2016). Concomitant EGFR mutations/ALK rearrangements: beyond a simple dual target. Transl Lung Cancer Res.

[44] Fan T, Song YJ, Liu XL (2016). Adenocarcinoma of the lung with concomitant ALK fusion gene and EGFR gene mutation: a case report and literature review. Mol Clin Oncol.

[45] Zhou J, Zheng J, Zhao J, Sheng Y, Ding W, Zhou J (2015). Poor response to gefitinib in lung adenocarcinoma with concomitant epidermal growth factor receptor mutation and anaplastic lymphoma kinase rearrangement. Thorac Cancer.

[46] Rossing HH, Grauslund M, Urbanska EM, Melchior LC, Rask CK, Costa JC, Skov BG, Sørensen JB, Santoni-Rugiu E (2013). Concomitant occurrence of EGFR (epidermal growth factor receptor) and KRAS (V-Ki-ras2 Kirsten rat sarcoma viral oncogene homolog) mutations in an ALK (anaplastic lymphoma kinase)-positive lung adenocarcinoma patient with acquired resistance to crizotinib: a case report. BMC Res Notes.

[47] Passaro A, Barberis M, Catania C, Pessina S, de Marinis F (2015). Concomitant ALK translocation and other non-EGFR gene in NSCLC: knowledge in the making. Ann Oncol.

[48] Lee T, Lee B, Choi YL, Han J, Ahn MJ, Um SW (2016). Non-small cell lung cancer with concomitant EGFR, KRAS, and ALK mutation: clinicopathologic features of 12 cases. J Pathol Transl Med.

[49] Ulivi P, Chiadini E, Dazzi C, Dubini A, Costantini M, Medri L, Puccetti M, Capelli L, Calistri D, Verlicchi A, Gamboni A, Papi M, Mariotti M (2016). Nonsquamous, non-small-cell lung cancer patients who carry a double mutation of EGFR, EML4-ALK or KRAS: frequency, clinical-pathological characteristics, and response to therapy. Clin Lung Cancer.

[50] Sahnane N, Frattini M, Bernasconi B, Zappa F, Schiavone G, Wannesson L, Antonelli P, Balzarini P, Sessa F, Mazzucchelli L, Tibiletti MG, Martin V (2016). EGFR and KRAS mutations in ALK-positive lung adenocarcinomas: biological and clinical effect. Clin Lung Cancer.

[51] Rossi G, Baldi L, Barbieri F, Bertolini F, Tiseo M (2015). Concomitant EGFR and KRAS mutations in ALK-rearranged lung cancer. Ann Oncol.

[52] Guibert N, Barlesi F, Descourt R, Léna H, Besse B, Beau-Faller M, Mosser J, Pichon E, Merlio JP, Ouafik L, Guichard F, Mastroianni B, Moreau L (2017). Characteristics and outcomes of patients with lung cancer harboring multiple molecular alterations: results from the IFCT study biomarkers France. J Thorac Oncol.

[53] Romanidou O, Landi L, Cappuzzo F, Califano R (2016). Overcoming resistance to first/second generation epidermal growth factor receptor tyrosine kinase inhibitors and ALK inhibitors in oncogene-addicted advanced non-small cell lung cancer. Ther Adv Med Oncol.

[54] Mujoomdar A, Austin JH, Malhotra R, Powell CA, Pearson GD, Shiau MC, Raftopoulos H (2007). Clinical predictors of metastatic disease to the brain from non-small cell lung carcinoma: primary tumor size, cell type, and lymph node metastases. Radiology.

[55] Andrews DW, Scott CB, Sperduto PW, Flanders AE, Gaspar LE, Schell MC, Werner-Wasik M, Demas W, Ryu J, Bahary JP, Souhami L, Rotman M, Mehta MP (2004). Whole brain radiation therapy with or without stereotactic radiosurgery boost for patients with one to three brain metastases: phase III results of the RTOG 9508 randomised trial. Lancet.

[56] Dempke WC, Edvardsen K, Lu S, Reinmuth N, Reck M, Inoue A (2015). Brain metastases in NSCLC- Are TKIs changing the treatment strategy. Anticancer Res.

[57] D’Antonio C, Passaro A, Gori B, Del Signore E, Migliorino MR, Ricciardi S, Fulvi A, de Marinis F (2014). Bone and brain metastases in lung cancer: recent advances in therapeutic strategies. Ther Adv Med Oncol.

[58] Proto C, Imbimbo M, Gallucci R, Brissa A, Signorelli D, Vitali M, Macerelli M, Corrao G, Ganzinelli M, Greco FG, Garassino MC, Lo Russo G (2016). Epidermal growth factor receptor tyrosine kinase inhibitors for the treatment of central nervous system metastases from non-small cell lung cancer: the present and the future. Transl Lung Cancer Res.

